# Physiological Interpretation of the Lactate to Pyruvate AUC Ratio for Hyperpolarized [1‐^13^C]‐Pyruvate Studies

**DOI:** 10.1002/mrm.70309

**Published:** 2026-02-27

**Authors:** Ryan T. Boyce, Collin J. Harlan, Qing Wang, Christopher M. Walker, Stephen Y. Lai, Matthew E. Merritt, James A. Bankson

**Affiliations:** ^1^ Department of Imaging Physics The University of Texas M.D. Anderson Cancer Center Houston Texas USA; ^2^ Department of Physics University of Houston Houston Texas USA; ^3^ The University of Texas MD Anderson Cancer Center UT Health Houston Graduate School of Biomedical Sciences Houston Texas USA; ^4^ Department of Head and Neck Surgery The University of Texas M.D. Anderson Cancer Center Houston Texas USA; ^5^ Department of Biochemistry and Molecular Biology University of Florida College of Medicine Gainesville Florida USA

**Keywords:** hyperpolarized pyruvate, kinetic modeling, tumor metabolism

## Abstract

**Purpose:**

To explore the relationship between potentially rate‐limiting steps in lactate production and the lactate area‐under‐the‐curve (AUC) to pyruvate AUC ratio for analysis of hyperpolarized (HP) [1‐^13^C]‐pyruvate data.

**Theory and Methods:**

Simplifying assumptions are introduced to a pharmacokinetic (PK) model with three physical compartments and two chemical pools to write the AUC ratio in terms of model parameters. Synthetic time curves are used to test sensitivity of the AUC ratio and model parameters to different physiological conditions and acquisition parameters. The simplified model is used to analyze data from patients with head and neck squamous cell carcinoma and determine the rate‐limiting step in lactate production.

**Results:**

The simplified model leads to an expression that explicitly demonstrates the relationship between PK model parameters and the AUC ratio. The AUC ratio depends most strongly on intracellular metabolism when pyruvate signal predominantly arises from intracellular space. Simulations confirm that the parameterized AUC ratio can be used to correct for AUC ratio sensitivity to acquisition parameters such as TE. In patient data, the proposed analysis most commonly identified *k*_*ecP*_ as the predominant rate limiting step in lactate production.

**Conclusion:**

Changes observed in the AUC ratio may be driven by changes in pyruvate extravasation, transport into the cell, and/or intracellular metabolism. The proposed model permits parametric representation of the AUC ratio, identification of rate‐limiting steps in lactate production, and correction for differences induced by acquisition parameters.

## 1 Introduction

Hyperpolarized (HP) [1‐^13^C]‐pyruvate MRI has found success in preclinical [1, 2, 3, 4, 5] and clinical [6, 7, 8] studies as a method for detecting early tumor response to treatment across several types of cancer. The successful translation of HP pyruvate MRI depends on robust quantification that faithfully represents clinically relevant parameters.

Aerobic glycolysis, or lactate production under normoxic conditions, is characteristic of many cancers [9]. Positioned at a metabolic crossroads, HP [1‐^13^C]‐pyruvate can be used to visualize and characterize aerobic glycolysis. After injection, a bolus of HP pyruvate travels through vasculature, then extravasates and must cross the cell membrane before exposure to intracellular enzymes that mediate its conversion into lactate. Uptake and intracellular metabolism of pyruvate are mediated by monocarboxylate transporters (primarily MCT1) and lactate dehydrogenase A (LDHA) respectively, both of which are overexpressed in many tumors [10, 11]. Finally, excess lactate is exported via MCT4, resulting in the acidification of the tumor microenvironment. Depending on disease state, extravasation from vasculature, transport across the cell membrane, and intracellular metabolism of pyruvate may play varying roles in the evolution of signals from HP pyruvate and lactate and must be considered when interpreting HP pyruvate signal.

Pharmacokinetic (PK) modeling describes the movement of the HP ^13^C label through the biological environment. The most common model is a precursor‐product model in which observed HP pyruvate is assumed to be in immediate chemical contact with LDHA. Models which separately track metabolites through more than one physical compartment have been previously used to account for the effects of pyruvate perfusion and uptake on lactate production [12]. While models with more parameters provide more information about the system, the error in fitted parameters increases with model complexity [13]. Additionally, fitted parameters are difficult to compare between studies because there is currently no universally accepted model [14] and parameters from different models have different physiological interpretations.

Individual metabolite curves are often summarized with AUC maps as an alternative to PK modeling, however this approach is not quantitative since MRI signal amplitudes are uncalibrated. The lactate to pyruvate area‐under‐the‐curve (AUC) ratio normalizes the lactate AUC map, resulting in a semi‐quantitative surrogate for lactate production and tumor metabolism. In current literature, the AUC ratio is as common as PK modeling [14] and correlates with intracellular pyruvate metabolism under certain conditions [15, 16]. However, because the AUC ratio is influenced by many factors, it is not straightforward to isolate the influence of intracellular metabolism on the observed AUC ratio. We seek to develop a framework to enhance interpretation of the AUC ratio in terms of PK parameters. To do so, we use a simplified kinetic model to derive a parameterized expression for the AUC ratio. The expression is simplified for three cases in which intravascular pyruvate, extravascular/extracellular pyruvate, or intracellular pyruvate represent dominant sources of HP signal, revealing differing dependencies on model parameters. We describe a method to identify the relative contributions of the three HP pyruvate pools for improved physiological interpretation of the AUC ratio.

## 2 Theory

### 2.1 Full Three‐Compartment Pharmacokinetic Model

**FIGURE 1:**
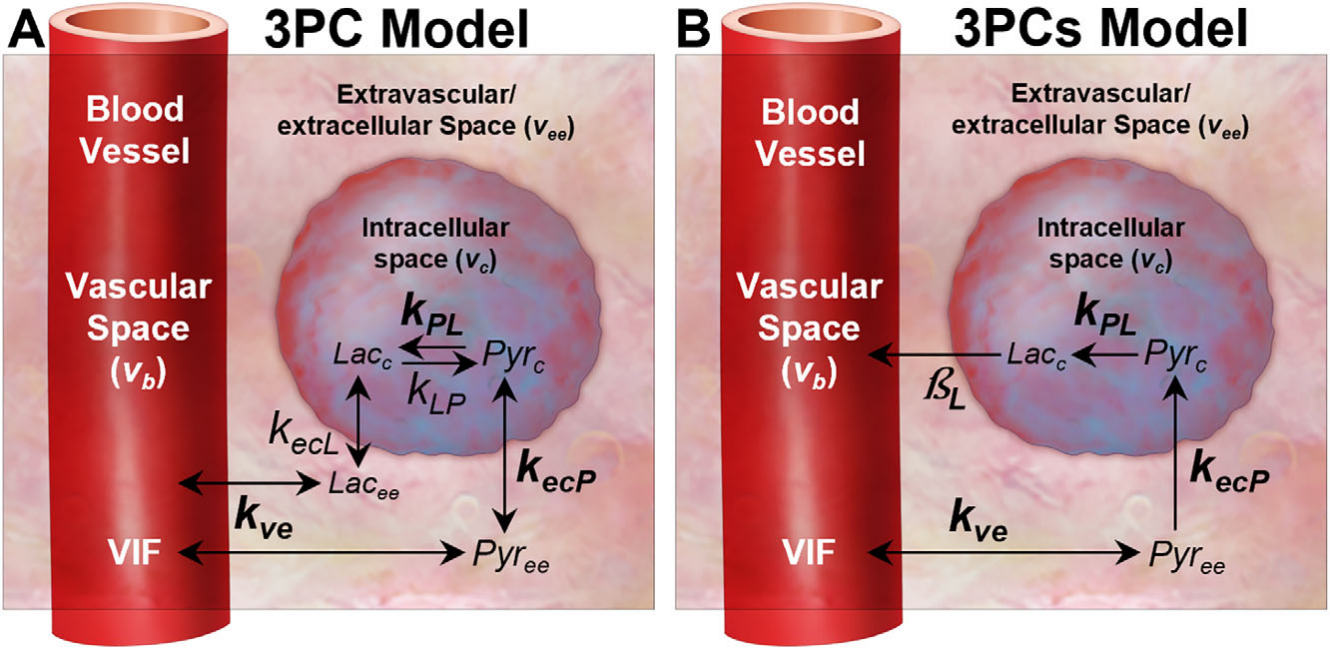
Graphic depiction of the full (3PC) and simplified (3PCs) three‐physical‐compartment models. Each model tracks the concentration of HP pyruvate and lactate moving through intravascular ($v_{b}$), extravascular/extracellular ($v_{\mathrm{ee}}$), and intracellular ($v_{c}$) volumes, assuming the chemical conversion of pyruvate to lactate $\left(k_{\mathrm{PL}}\right)$ only takes place in the intracellular compartment where the requisite enzymes are present. $k_{\mathrm{ve}}$ is the linear rate constant for extravasation of HP pyruvate, and $k_{\mathrm{ecP}}$ reflects is the rate of pyruvate transport across the cell membrane. Lactate flux across the cell membrane ($k_{\mathrm{ecL}}$) is only modeled in the 3PC model and replaced by a heuristic term for lactate efflux term ($\beta_{L}$) in the 3PCs model. The vascular input function (VIF) represents the measured HP pyruvate in the blood supply. Adapted from Boyce R, Harlan C, Wang Q, et al. Physiological Interpretation of the AUC Ratio for Hyperpolarized [1‐^13^C]‐Pyruvate. Abstract presented at: International Society for Magnetic Resonance in Medicine Annual Meeting & Exhibition; May 6, 2024; Singapore. 5079.

A dose of HP pyruvate is delivered intravenously and must cross the vascular endothelium and the cell membrane before exchanging with lactate in the cytoplasm. A three‐physical‐compartment (3PC) PK model describes pyruvate and lactate dynamics through intravascular, extravascular/extracellular, and intracellular spaces (Figure 1A). While all models are approximations, the 3PC model provides the most information about the flux and label exchange between compartments. Flux between compartments is described by Fick's law as the product of a constant and the concentration difference between compartments. In this model, flux across the vascular endothelium is given by $k_{\mathrm{ve}}$ (s^−1^) while flux across the cell membrane is described by $k_{\mathrm{ecP}}$ (s^−1^) for pyruvate and $k_{\mathrm{ecL}}$ (s^−1^) for lactate, assuming MCTs are not saturated by HP pyruvate.

Inside the cell, LDHA catalyzes the reversible chemical conversion of HP pyruvate into lactate. Chemical conversion is modeled using first‐order kinetics, with the conversion of HP pyruvate into lactate described by the apparent rate constant $k_{\mathrm{PL}}$ (s^−1^) and the reverse reaction described by $k_{\mathrm{LP}}$ (s^−1^). MR signal is proportional to ^13^C nuclei, determined by the volume fraction and concentration of the chemical in a given compartment. Total signal is the sum across compartments. The differential equations and parameterized AUC ratio for the full 3PC model can be calculated using the following process and is included in Supporting Information Equation (S1–S6).

### 2.2 Parameterized Representation of the AUC Ratio Using a Simplified Three‐Compartment Model

The AUC ratio can be concisely parametrized in terms of PK parameters with the use of a simplified model (Figure 1B) which still accounts for the effects of pyruvate extravasation ($k_{\mathrm{ve}}$), transport ($k_{\mathrm{ecP}}$), and metabolism ($k_{\mathrm{PL}}$). The simplified three‐physical‐compartment (3PCs) model tracks the intravascular (*iv*), extravascular/extracellular (*ee*), and intracellular (*c*) compartments, but HP pyruvate efflux from the cell is assumed to be negligible during the short time course of an HP experiment. Similarly, we assume that the rate of intracellular chemical conversion from lactate back to pyruvate is negligible. Since the spin‐labeled lactate signal is quickly depleted by *T*_1_ relaxation and excitation losses, we assume that the HP lactate signal arises primarily from intracellular space [17]. Because reverse flux and HP lactate export/intravasation has been eliminated in this model, an additional term $\left(\beta_{L}\right)$ is included to permit attenuation or loss of intracellular HP lactate spins from the simplified system. Longitudinal magnetization is continuously decaying toward thermal equilibrium due to *T*_1_ relaxation ($R_{1,\mathrm{Pyr}}$ and $R_{1,\mathrm{Lac}}$) and is depleted by RF excitations with flip angles of $\theta_{\mathrm{Pyr}}$ and $\theta_{\mathrm{Lac}}$ for pyruvate and lactate, respectively. Following the work of Kazan et al. [18], we assume that no lactate signal is found in the VIF. The resulting system of differential equations for the 3PCs model is decoupled.

(1) $$\begin{array}{cc} \frac{\partial }{\partial t}\left[\mathrm{Py}r_{\mathrm{ee}}\left(t\right)\right] & =-\left(\frac{k_{\mathrm{ve}}}{v_{\mathrm{ee}}}+\frac{k_{\mathrm{ecP}}}{v_{\mathrm{ee}}}+R_{1,\mathrm{Pyr}}+\frac{1-\cos\;\theta_{\mathrm{Pyr}}}{\mathrm{TR}}\right)\left[\mathrm{Py}r_{\mathrm{ee}}\left(t\right)\right]\\ & \;+\frac{k_{\mathrm{ve}}}{v_{\mathrm{ee}}}\left[\mathrm{Py}r_{\mathrm{iv}}\left(t\right)\right]=\alpha_{\mathrm{Pee}}\left[\mathrm{Py}r_{\mathrm{ee}}\left(t\right)\right]+\frac{k_{\mathrm{ve}}}{v_{\mathrm{ee}}}\left[\mathrm{Py}r_{\mathrm{iv}}\left(t\right)\right] \end{array}$$

(2) $$\begin{array}{cc} \frac{\partial }{\partial t}\left[\mathrm{Py}r_{c}\left(t\right)\right] & =\frac{k_{\mathrm{ecP}}}{v_{c}}\left[\mathrm{Py}r_{\mathrm{ee}}\left(t\right)\right]-\left(k_{\mathrm{PL}}+R_{1,\mathrm{Pyr}}+\frac{1-\cos\;\theta_{\mathrm{Pyr}}}{\mathrm{TR}}\right)\left[\mathrm{Py}r_{c}\left(t\right)\right]\\ & \;=\frac{k_{\mathrm{ecP}}}{v_{c}}\left[\mathrm{Py}r_{\mathrm{ee}}\left(t\right)\right]+\alpha_{\mathrm{Pc}}\left[\mathrm{Py}r_{c}\left(t\right)\right] \end{array}$$

(3) $$\begin{array}{cc} \frac{\partial }{\partial t}\left[\mathrm{La}c_{c}\left(t\right)\right] & =k_{\mathrm{PL}}\left[\mathrm{Py}r_{c}\left(t\right)\right]-\left(R_{1,\mathrm{Lac}}+\frac{1-\cos\;\theta_{\mathrm{Lac}}}{\mathrm{TR}}+\beta_{L}\right)\left[\mathrm{La}c_{c}\left(t\right)\right]\\ & \;=k_{\mathrm{PL}}\left[\mathrm{Py}r_{c}\left(t\right)\right]+\alpha_{\mathrm{Lc}}\left[\mathrm{La}c_{c}\left(t\right)\right] \end{array}$$

Taking the Laplace transform of Equations ((1), (2), (3)) yields

(4) $$\begin{array}{c} s\cdot \bar{\mathrm{Py}r_{\mathrm{ee}}}\left(s\right)-\left[\mathrm{Py}r_{\mathrm{ee}}\left(t=0\right)\right]=\alpha_{\mathrm{Pee}}\bar{\mathrm{Py}r_{\mathrm{ee}}}\left(s\right)+\frac{k_{\mathrm{ve}}}{v_{\mathrm{ee}}}\bar{\mathrm{Py}r_{\mathrm{iv}}}\left(s\right) \end{array}$$

(5) $$\begin{array}{c} \;s\cdot \bar{\mathrm{Py}r_{c}}\left(s\right)-\left[\mathrm{Py}r_{c}\left(t=0\right)\right]=\frac{k_{\mathrm{ecP}}}{v_{c}}\bar{\mathrm{Py}r_{\mathrm{ee}}}\left(s\right)+\alpha_{\mathrm{Pc}}\bar{\mathrm{Py}r_{c}}\left(s\right) \end{array}$$

(6) $$\begin{array}{c} s\cdot \bar{\mathrm{La}c_{c}}\left(s\right)-\left[\mathrm{La}c_{c}\left(t=0\right)\right]=k_{\mathrm{PL}}\bar{\mathrm{Py}r_{c}}\left(s\right)+\alpha_{\mathrm{Lc}}\bar{\mathrm{La}c_{c}}\left(s\right) \end{array}$$

where *s* is the Laplace variable. Assuming signal acquisition begins before the HP bolus is injected, $\left[{\mathrm{Pyr}}_{\mathrm{ee}}\left(t=0\right)\right]=\left[{\mathrm{Pyr}}_{c}\left(t=0\right)\right]=\left[{\mathrm{Lac}}_{c}\left(t=0\right)\right]=0$. Examining the AUCs for each metabolite concentration further simplifies Equations ((4), (5), (6)) because the AUC is equivalent to evaluating the value of the transformed functions at *s* = 0, and therefore the left‐hand side of each equation vanishes. Then, each AUC is proportional to the AUC for $\left[\mathrm{Py}r_{\mathrm{iv}}\left(t\right)\right]$, which is the VIF.

(7) $$\begin{array}{c} \bar{\mathrm{Py}r_{\mathrm{ee}}}\left(s=0\right)=\frac{-k_{\mathrm{ve}}}{\alpha_{\mathrm{Pee}}v_{\mathrm{ee}}}\bar{\mathrm{Py}r_{\mathrm{iv}}}\left(s=0\right) \end{array}$$

(8) $$\begin{array}{c} \;\bar{\mathrm{Py}r_{c}}\left(s=0\right)=\frac{k_{\mathrm{ecP}}k_{\mathrm{ve}}}{\alpha_{\mathrm{Pc}}v_{c}\alpha_{\mathrm{Pee}}v_{\mathrm{ee}}}\bar{\mathrm{Py}r_{\mathrm{iv}}}\left(s=0\right) \end{array}$$

(9) $$\begin{array}{c} \;\bar{\mathrm{La}c_{c}}\left(s=0\right)=\frac{-k_{\mathrm{PL}}k_{\mathrm{ecP}}k_{\mathrm{ve}}}{\alpha_{\mathrm{Lc}}\alpha_{\mathrm{Pc}}v_{c}\alpha_{\mathrm{Pee}}v_{\mathrm{ee}}}\bar{\mathrm{Py}r_{\mathrm{iv}}}\left(s=0\right) \end{array}$$

The signal AUC of each pool depends on the number of spins present as well as the flip angle, echo time (TE), and $T_{2}^{*}$ of the pool in that compartment. Thus,

(10) $$\begin{array}{c} \;\mathrm{AUC}\left(\mathrm{Py}r_{\mathrm{iv}}\right)=v_{b}\bar{\mathrm{Py}r_{\mathrm{iv}}}\left(s=0\right)\cdot \sin\;\theta_{\mathrm{Pyr}}\cdot \exp\left(-\mathrm{TE}/T_{2,\mathrm{Pv}}^{*}\right) \end{array}$$

(11) $$\begin{array}{c} \;\mathrm{AUC}\left(\mathrm{Py}r_{\mathrm{ee}}\right)=v_{\mathrm{ee}}\bar{\mathrm{Py}r_{\mathrm{ee}}}\left(s=0\right)\cdot \sin\;\theta_{\mathrm{Pyr}}\cdot \exp\left(-\mathrm{TE}/T_{2,\mathrm{Pe}}^{*}\right) \end{array}$$

(12) $$\begin{array}{c} \;\mathrm{AUC}\left(\mathrm{Py}r_{c}\right)=v_{c}\bar{\mathrm{Py}r_{c}}\left(s=0\right)\cdot \sin\;\theta_{\mathrm{Pyr}}\cdot \exp\left(-\mathrm{TE}/T_{2,\mathrm{Pc}}^{*}\right) \end{array}$$

(13) $$\begin{array}{c} \mathrm{AUC}\left(\mathrm{La}c_{c}\right)=v_{c}\bar{\mathrm{La}c_{c}}\left(s=0\right)\cdot \sin\;\theta_{\mathrm{Lac}}\cdot \exp\left(-\mathrm{TE}/T_{2,\mathrm{Lc}}^{*}\right) \end{array}$$

and the AUC ratio can be written as:

(14) $$\begin{array}{c} \frac{\mathrm{AU}C_{\mathrm{Lac}}}{\mathrm{AU}C_{\mathrm{Pyr}}}=\frac{\mathrm{AUC}\left(\mathrm{La}c_{c}\right)}{\mathrm{AUC}\left(\mathrm{Py}r_{c}\right)+\mathrm{AUC}\left(\mathrm{Py}r_{\mathrm{ee}}\right)+\mathrm{AUC}\left(\mathrm{Py}r_{\mathrm{iv}}\right)} \end{array}$$

Substitution of Equations ((7), (8), (9)) and Equations ((10), (11), (12), (13)) into Equation (14) gives

(15) $$\begin{array}{c} \;\frac{\mathrm{AU}C_{\mathrm{Lac}}}{\mathrm{AU}C_{\mathrm{Pyr}}}=\frac{\sin\;\theta_{\mathrm{Lac}}}{\sin\;\theta_{\mathrm{Pyr}}}\cdot \frac{k_{\mathrm{ve}}k_{\mathrm{ecP}}k_{\mathrm{PL}}e^{-\mathrm{TE}/T_{2,\mathrm{Lc}}^{*}}}{v_{\mathrm{ee}}\alpha_{\mathrm{Lc}}\alpha_{\mathrm{Pc}}\left(k_{\mathrm{ve}}e^{-\mathrm{TE}/T_{2,\mathrm{Pee}}^{*}}-\alpha_{\mathrm{Pee}}v_{b}e^{-\mathrm{TE}/T_{2,\mathrm{Pv}}^{*}}\right)-\alpha_{\mathrm{Lc}}k_{\mathrm{ve}}k_{\mathrm{ecP}}e^{-\mathrm{TE}/T_{2,\mathrm{Pc}}^{*}}} \end{array}$$

If the pyruvate signal time curves are dominated by intracellular spins, $v_{b}\bar{\mathrm{Py}r_{\mathrm{iv}}}\left(s=0\right)+v_{\mathrm{ee}}\bar{\mathrm{Py}r_{\mathrm{ee}}}\left(s=0\right)+v_{c}\bar{\mathrm{Py}r_{c}}\left(s=0\right)\approx v_{c}\bar{\mathrm{Py}r_{c}}\left(s=0\right)$, or equivalently $k_{\mathrm{ve}}k_{\mathrm{ecP}}\gg v_{\mathrm{ee}}\left(\alpha_{\mathrm{Pee}}\alpha_{\mathrm{Pc}}v_{b}-\alpha_{\mathrm{Pc}}k_{\mathrm{ve}}\right)$, the first and second terms in the denominator become negligible and, neglecting $\mathrm{TE}/T_{2}^{*}$ effects and defining $\Theta =\sin\;\theta_{\mathrm{Lac}}/\sin\;\theta_{\mathrm{Pyr}}$, the expression for the AUC ratio becomes

(16) $$\begin{array}{c} \;\frac{\mathrm{AU}C_{\mathrm{Lac}}}{\mathrm{AU}C_{\mathrm{Pyr}}}=\Theta \cdot \frac{-k_{\mathrm{PL}}}{\alpha_{\mathrm{Lc}}} \end{array}$$

which shows that under these conditions, the AUC ratio is proportional to the apparent rate for conversion of HP pyruvate into lactate and inversely proportional with the rate of intracellular lactate signal loss.

If instead the pyruvate signal time curves are dominated by spins in the extravascular/extracellular compartment, $v_{b}\bar{\mathrm{Py}r_{\mathrm{iv}}}\left(s=0\right)+v_{\mathrm{ee}}\bar{\mathrm{Py}r_{\mathrm{ee}}}\left(s=0\right)+v_{c}\bar{\mathrm{Py}r_{c}}\left(s=0\right)\approx v_{\mathrm{ee}}\bar{\mathrm{Py}r_{\mathrm{ee}}}\left(s=0\right)$, or $-v_{\mathrm{ee}}\alpha_{\mathrm{Pc}}k_{\mathrm{ve}}\gg v_{\mathrm{ee}}\alpha_{\mathrm{Pee}}\alpha_{\mathrm{Pc}}v_{b}+k_{\mathrm{ve}}k_{\mathrm{ecP}}$, then the first term in the denominator of Equation (15) dominates and the expression for the AUC ratio can be written as

(17) $$\begin{array}{c} \;\frac{\mathrm{AU}C_{\mathrm{Lac}}}{\mathrm{AU}C_{\mathrm{Pyr}}}=\Theta \cdot \frac{k_{\mathrm{ecP}}}{v_{\mathrm{ee}}\alpha_{\mathrm{Pc}}}\cdot \left(\frac{k_{\mathrm{PL}}}{\alpha_{\mathrm{Lc}}}\right) \end{array}$$

In the case that extravascular/extracellular pyruvate signal dominates, the AUC ratio gains a sensitivity to $k_{\mathrm{ecP}}$, the extracellular/extravascular volume fraction, and the rate of signal loss of intracellular pyruvate. It still depends on $k_{\mathrm{PL}}$ and lactate signal loss, though these are no longer the only important physiological factors. Since the rate of intracellular pyruvate loss ($\alpha_{\mathrm{Pc}}$) also depends on $k_{\mathrm{PL}}$, under these conditions, the AUC ratio is not strictly proportional to $k_{\mathrm{PL}}$, but is proportional to $k_{\mathrm{ecP}}$.

Moving back one step further in the process, we finally consider the case in which pyruvate signal is dominated by spins in the vascular compartment, when $v_{b}\bar{\mathrm{Py}r_{\mathrm{iv}}}\left(s=0\right)+v_{\mathrm{ee}}\bar{\mathrm{Py}r_{\mathrm{ee}}}\left(s=0\right)+v_{c}\bar{\mathrm{Py}r_{c}}\left(s=0\right)\approx v_{b}\bar{\mathrm{Py}r_{\mathrm{iv}}}\left(s=0\right)$ or $v_{\mathrm{ee}}\alpha_{\mathrm{Pee}}\alpha_{\mathrm{Pc}}v_{b}\gg k_{\mathrm{ve}}k_{\mathrm{ecP}}-v_{\mathrm{ee}}\alpha_{\mathrm{Pc}}k_{\mathrm{ve}}$. Under these conditions, the AUC ratio can be written as

(18) $$\begin{array}{c} \;\frac{\mathrm{AU}C_{\mathrm{Lac}}}{\mathrm{AU}C_{\mathrm{Pyr}}}=\Theta \cdot \frac{-k_{\mathrm{ve}}}{v_{b}\alpha_{\mathrm{Pee}}}\cdot \left(\frac{k_{\mathrm{ecP}}}{v_{\mathrm{ee}}\alpha_{\mathrm{Pc}}}\cdot \frac{k_{\mathrm{PL}}}{\alpha_{\mathrm{Lc}}}\right) \end{array}$$

In addition to the parameters from Equation (17), this is expression depends on the rate of pyruvate extravasation, on the intravascular volume fraction, and on the rate of pyruvate signal loss from the extravascular/extracellular compartment. Since extravascular/extracellular pyruvate signal loss $\left(\alpha_{\mathrm{Pee}}\right)$ depends on both $k_{\mathrm{ve}}$ and $k_{\mathrm{ecP}}$, Equation (18) is not strictly proportional to any of the kinetic rate constants.

Taken together, Equations ((16), (17), (18)) indicate that the AUC ratio is only strictly proportional to intracellular pyruvate metabolism in the case that intracellular spins dominate the pyruvate signal. When pyruvate signal from other compartments dominate, then the AUC ratio will become more dependent on other potentially rate‐limiting steps in lactate production. As the pyruvate signal is less dominated by intracellular spins, the AUC ratio functions less as a specific biomarker of intracellular metabolism because it becomes more dependent on other factors.

## 3 Methods

### 3.1 Generation of Synthetic Data for Pharmacokinetic Analyses

The ability of the simplified 3PCs model to fit time curves from the full 3PC model was evaluated by fitting the 3PCs model to synthetic data from the full 3PC model for the three sets of PK parameters in Table 1A. These parameter sets represent physiological conditions where the HP pyruvate signal predominantly arises from intravascular, extravascular/extracellular, and intracellular spaces. $k_{\mathrm{PL}}$, $k_{\mathrm{ecP}}$, $k_{\mathrm{ve}}$, $\beta_{L}$, and the VIF amplitude scaling constant for the 3PCs model were fit to synthetic data generated using the 3PC model. Models were implemented in MATLAB R2023a (The MathWorks, Natick, MA).

**TABLE 1:** Model parameters used for generating the synthetic datasets used in simulations.

	(A) 3PC model parameters			(B) 3PCs model parameters		
	Pyr_iv_ dominates	Pyr_ee_ dominates	Pyr_c_ dominates	Pyr_iv_ dominates	Pyr_ee_ dominates	Pyr_c_ dominates
*k*_*PL*_ (s^−1^)	0.5	0.5	0.2	0.5645	0.5371	0.2061
*k*_*LP*_ (s^−1^)	0.02	0.02	0.0008	—	—	—
*k*_*ecP*_ (s^−1^)	0.03	0.03	0.2	0.0253	0.0293	0.1211
*k*_*ecL*_ (s^−1^)	0.03	0.03	0.2	—	—	—
*k*_*ve*_ (s^−1^)	0.011	0.066	0.08	0.0113	0.0566	0.1656
*ß*_*L*_ (s^−1^)	—	—	—	0.0095	0.0393	0.078
*v*_*b*_	0.15	0.1	0.02	0.15	0.1	0.02
*v*_*ee*_	0.425	0.45	0.196	0.425	0.45	0.196
*v*_*c*_	0.425	0.45	0.784	0.425	0.45	0.784
Pyr_iv_/Pyr	60.6%	24.6%	7.1%	61.0%	27.1%	5.0%
Pyr_ee_/Pyr	34.9%	67.1%	30.3%	35.6%	65.5%	27.3%
Pyr_c_/Pyr	4.5%	8.3%	62.7%	3.5%	7.4%	67.7%

*Note*: Pyr_iv_/Pyr, Pyr_ee_/Pyr, and Pyr_c_/Pyr reflect the percentage of the total pyruvate signal that spins from the intravascular, extravascular/extracellular, or intracellular compartments contribute. Parameters that are not applicable to a particular model are marked with a dash.

For fitting, the MATLAB lsqnonlin solver was used with an error function defined as a simple residual of pyruvate and lactate signals normalized by their maxima to weight them equally. Initial seeds for the algorithm were drawn uniformly at random over the interval [0, 1]. Fits were repeated 500 times each with fresh seeds to avoid local minima in the residual. The VIF was assumed to follow a gamma variate function [19].

Because HP magnetization is inherently time‐dependent, signal curves are heavily influenced by relaxation properties of the label [13, 20]. *T*_1_s were assumed to be 43 s for pyruvate and 33 s for lactate. When evaluating echo time effects, intravascular pyruvate was assumed to have *T*_2_* = 100 ms, intracellular and extravascular/extracellular pyruvate were both assumed to have *T*_2_* = 55 ms, and for intracellular lactate *T*_2_* = 33 ms was assumed [20]. Constant flip angles of 20° for pyruvate and 30° for lactate were used for both simulations and acquisitions.

### 3.2 Assessing the Sensitivity of the AUC Ratio to the Three Potentially Rate‐Limiting Steps in Lactate Production

Three sets of PK parameters were identified to yield kinetic curves that were dominated by intravascular, extravascular/extracellular, and intracellular pyruvate. These parameters, summarized in Table 1, were then used to generate synthetic data to assess the sensitivity of the AUC ratio to the three potentially rate‐limiting steps in lactate production. For each parameter set, one kinetic rate constant was varied at a time and the AUC ratio was calculated using Equation (15) to explore the relative impact of those parameters under varying physiological conditions.

### 3.3 Assessing the Sensitivity of Fitted Kinetic Rate Constants to Potentially Rate‐Limiting Steps in Lactate Production

Pyruvate extravasation (*k*_*ve*_), uptake (*k*_*ecP*_), and metabolism (*k*_*PL*_) all may be rate‐limiting steps in lactate production in different tissues and disease states. To assess the feasibility of quantifying a non‐rate‐limiting step, the 3PCs model was fitted to synthetic data generated from the three parameter sets listed in Table 1B with added zero‐mean Gaussian random noise. The standard deviation of the Gaussian noise distribution was chosen to achieve a target pyruvate peak SNR according to Equation (19). Gaussian noise was resampled and the kinetic analysis was repeated 100 times for estimation of the mean and standard deviation of fit parameter values as a function of SNR and parameter set. $k_{\mathrm{PL}}$, $k_{\mathrm{ecP}}$, $k_{\mathrm{ve}}$ and a VIF amplitude scaling constant were fit; all other parameters were assumed to be known.

(19) $$\begin{array}{c} \mathrm{SNR}=\frac{\max\left(\mathrm{Py}r_{\mathrm{tot}}\left(t\right)\right)}{\sigma_{\text{noise}}} \end{array}$$

### 3.4 Effect of Echo Time on Quantification of Lactate Production

Multi‐site trials for [1‐^13^C]‐pyruvate depend on reproducible metrics of lactate production, which may be hindered by inconsistent choices of acquisition parameters such as echo time (TE) at different centers [21]. The relationship between TE and quantification of lactate production was characterized by generating synthetic data over a range of TE values. The application of the 3PCs model and Equation (15) to correct the AUC ratio for any differences caused by variations in echo time was tested by traditional least squares fitting to noisy synthetic data and recalculation of Equation (15) with reference acquisition parameters. First, 50 sets of synthetic data were generated using the Pyr_ee_ dominant parameters in Table 1B and randomly sampled Gaussian noise to achieve a peak pyruvate SNR of 20. The “integrated” AUC ratio was calculated in the traditional way by summing under the curves. Next, the 3PCs model was fitted to the synthetic data and Equation (15) was used to calculate the model‐derived AUC ratio. The parameters required for fitting were *k*_*PL*_, *k*_*ecP*_, *k*_*ve*_, *v*_*b*_, *v*_*ee*_, *v*_*c*_, $\beta_{L}$, and a VIF amplitude scaling factor. Finally, the AUC ratio was corrected for TE effects by recalculating the model‐derived AUC ratio with a TE of 20 ms.

### 3.5 Determination of Dominant HP Pyruvate Pool

The sensitivity and specificity of the model with respect to classification of the largest source of pyruvate signal was evaluated in simulation by generating data from the three parameter sets in Table 1B. The 3PCs model was used to generate synthetic data and a controlled amount of noise was added as described above. The 3PCs model was then fit to noisy data using a method that minimizes both the residual between noisy synthetic data and model estimates [22] and the L2 norm of physiological model parameters (manuscript under review). The fit parameters were used to identify the relative amplitude of the intravascular, extravascular/extracellular, and intracellular HP pyruvate signal pools using the terms in the denominator of Equation (15). The analysis was repeated 50 times at each SNR level. Sensitivity was defined as the number of times the analysis correctly identified the dominant compartment normalized by the total number of time that compartment was truly dominant. Specificity was defined as the number of times the analysis correctly identified a compartment as not dominant normalized by the total number of times that compartment was truly not dominant.

### 3.6 Acquisition of ^1^H Patient Data

Data acquired prior to start of treatment from patients with squamous cell carcinoma (SCC) were analyzed for this study, including four datasets from three patients with oropharyngeal SCC at the base of the tongue and one dataset from a patient with cutaneous SCC. All scanning was carried out on a 3 T MR750 system (GE Healthcare, Waukesha WI, USA) as part of an IRB‐approved study. Patients with tumors at the base of tongue were positioned head‐first and supine in a ^13^C Helmholtz “clamshell” transmit coil (GE Healthcare, Waukesha WI, USA). For receive, two four‐element ^13^C “paddle” receive arrays were placed on both sides of the oral cavity. The patient with cutaneous SCC was scanned using a dual‐tuned ^1^H/^13^C head coil with eight ^13^C receive coils (Rapid MR International, Colombus OH, USA). To visualize tumor location, patients were first scanned with an axial *T*_2_‐weighted fast spin‐echo sequence with an echo time (TE) of 100 ms, a TR of 8199 ms and an echo train length of 18. A 256 × 256 acquisition matrix was used over a square FOV of 256 mm for an in‐plane spatial resolution of 1 × 1 mm. The slice thickness was 3.5 mm. Proton images were acquired through the body coil or the ^1^H transmit/receive channel of the head coil.

### 3.7 Acquisition of ^13^C Patient Data

A 250 mM solution of [1‐^13^C]‐pyruvate was prepared using a 5 T SPINlab hyperpolarizer (GE Healthcare, Waukesha, WI, USA). Approximately 1.44 g of [1‐^13^C]‐pyruvic acid was mixed with 15 mM electron paramagnetic agent (EPA) AH111501, sealed in a consumable fluid path, and loaded into the SPINlab system. After suitable buildup of polarization, the mixture was quickly dissolved with a 130°C sterile water, neutralized, and filtered. After confirmation that the pH, temperature, concentration of pyruvate, and concentration of EPA in the final solution met acceptance criteria, a dose of 0.43‐mL/kg from was administered at 5 mL/s followed by a 20 mL saline flush.

^13^C scanning was performed using spatially and spectrally selective excitations for pyruvate and lactate with an EPI readout [23]. Eight 15 mm slices were encoded with a matrix size of 16 × 16 over a 24 × 24 cm field of view. Constant flip angles of 20° for pyruvate and 30° for lactate were used for the dynamic acquisition [24]. A repetition time of 3 s was used with the acquisition lasting for 3 min. Data acquisition was initiated approximately 15 s before the start of the bolus injection of HP pyruvate. Magnetization was depleted by about 100 s after injection.

### 3.8 Pharmacokinetic Analysis of Patient Data

The 3PCs model and parameter‐minimized least squares method described above were used to fit $k_{\mathrm{PL}}$, $k_{\mathrm{ecP}}$, $k_{\mathrm{ve}}$, $v_{b}$, $v_{\mathrm{ee}}$, $v_{c}$, $\beta_{L}$, and the VIF amplitude scaling constant for each voxel. The VIF was identified from a voxel including the carotid artery. The peak SNR of each voxel was calculated according to Equation (19), with the standard deviation of the noise calculated from dynamic data beginning a few seconds after the HP magnetization was depleted.

The integrated AUC ratio for each voxel was calculated by summing over the observed lactate and pyruvate signal time curves and the model‐derived AUC ratio was calculated with Equation (15) from kinetic parameters fitted with the parameter‐minimized least squares approach. For the integrated AUC ratio, the dynamic time signals were truncated for the integral prior to bolus arrival and after the HP magnetization was fully depleted to minimize the included noise. The contribution of the three compartments to the overall pyruvate signal was mapped for the five in vivo acquisitions of patients with HNSCC. The analysis was carried out on a total of 74 voxels containing at least 30% tumor and with total ^13^C SNR greater than 10.

## 4 Results

### 4.1 The 3PCs Model Matches Curves Generated by the 3PC Model

**FIGURE 2:**
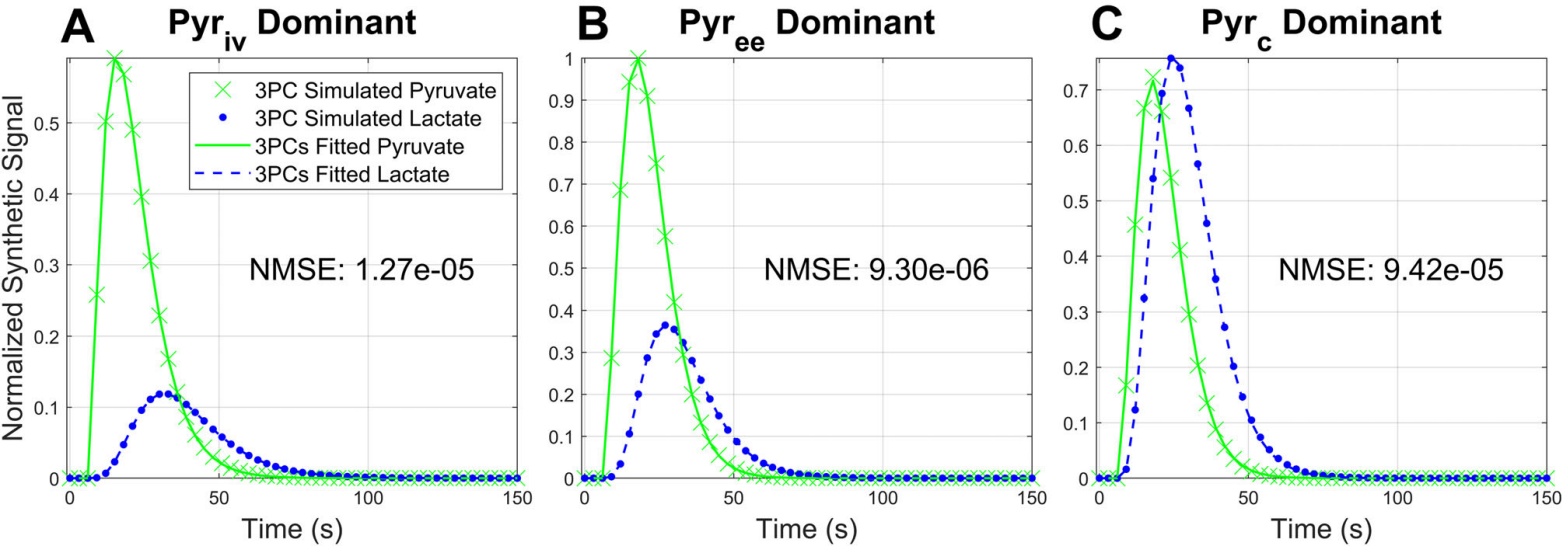
3PCs model fits 3PC simulated time curves for three representative sets of PK parameters (Table 1). Synthetic data curves were generated with the 3PC model for cases where the pyruvate signal was dominated by the intravascular (A), extravascular/extracellular (B), and intracellular compartments (C). For each case, the 3PCs model fit with very little error, indicating the simplifying assumptions in the 3PCs model do not significantly alter the fundamental shape of dynamic curves.

The 3PCs model recapitulated time curves from the 3PC model with very low error (Figure 2), demonstrating that the simplified model can yield curves that are numerically equivalent to the full model. The NMSE of for each of the three kinetic parameter sets examined was on the order of 10^−5^ or lower, which is much lower than the residual error that would be introduced by random noise in an in vivo acquisition. The 3PCs fitted rate constants (Table 1B) were not identical to their counterparts in the 3PC synthetic data but remained within a reasonable range and followed the same trends as the generating parameters.

### 4.2 Sensitivity of the AUC Ratio to Kinetic Rate Constants Depends on Dominant HP Pyruvate Pool

**FIGURE 3:**
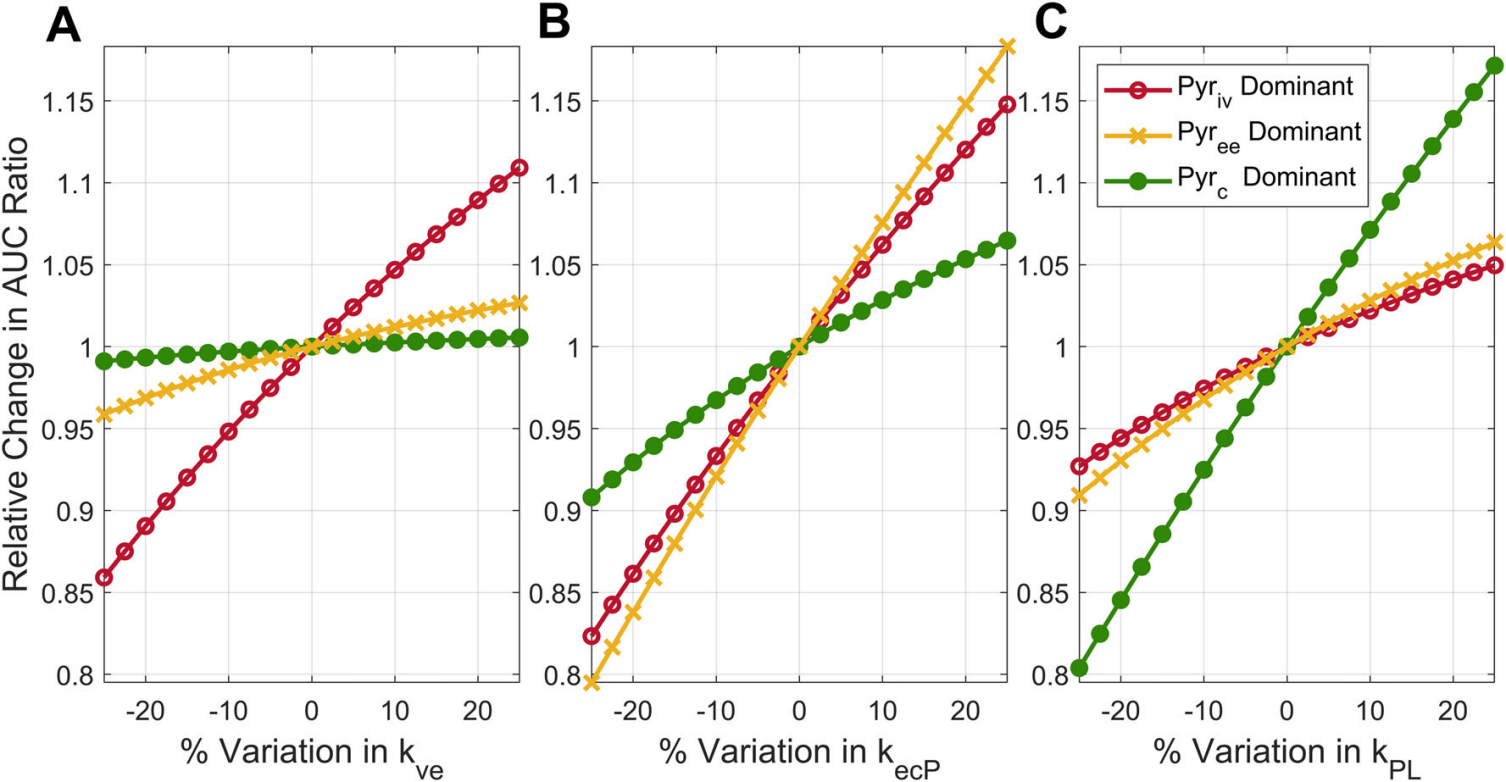
AUC ratio dependence on rate‐limiting model parameters and underlying physiology. The AUC ratio was calculated according to Equation (15) from 3PCs model parameters (Table 1) representing three cases where the pyruvate signal was dominated by spins in the intravascular (red curves with open circles), extravascular/extracellular (yellow curves with *x* symbols), and intracellular compartments (green curves with filled circles). For each regime, the sensitivity of the AUC ratio to changes in PK parameters was examined by varying one parameter and plotting the change in the calculated AUC ratio. When intravascular pyruvate dominates, varying $k_{\mathrm{ve}}$ and $k_{\mathrm{ecP}}$ resulted in the largest variations in the AUC ratio, as evidenced by the steep slope of the red curves in (A) and (B). When extravascular/extracellular pyruvate dominates, the AUC ratio was most sensitive to changes in $k_{\mathrm{ecP}}$. Only when intracellular pyruvate dominated was the AUC ratio most sensitive to changes in $k_{\mathrm{PL}}$ the rate of intracellular metabolism.

When pyruvate signal predominantly arose from intravascular space, the AUC ratio depended more strongly on pyruvate extravasation, $k_{\mathrm{ve}}$, and transport, $k_{\mathrm{ecP}}$, than on $k_{\mathrm{PL}}$, as evidenced by the larger relative slope of the red (open‐circle) curves in Figure 3. When pyruvate signal predominantly arose from extravascular/extracellular space, the AUC ratio depended most strongly on $k_{\mathrm{ecP}}$, the rate of pyruvate uptake into the cell (Figure 3B). The AUC ratio was most closely associated with $k_{\mathrm{PL}}$ when the dominant source of pyruvate signal arose from intracellular space (Figure 3C).

### 4.3 Precision of Kinetic Rate Constants Depends on Dominant HP Pyruvate Pool

**FIGURE 4:**
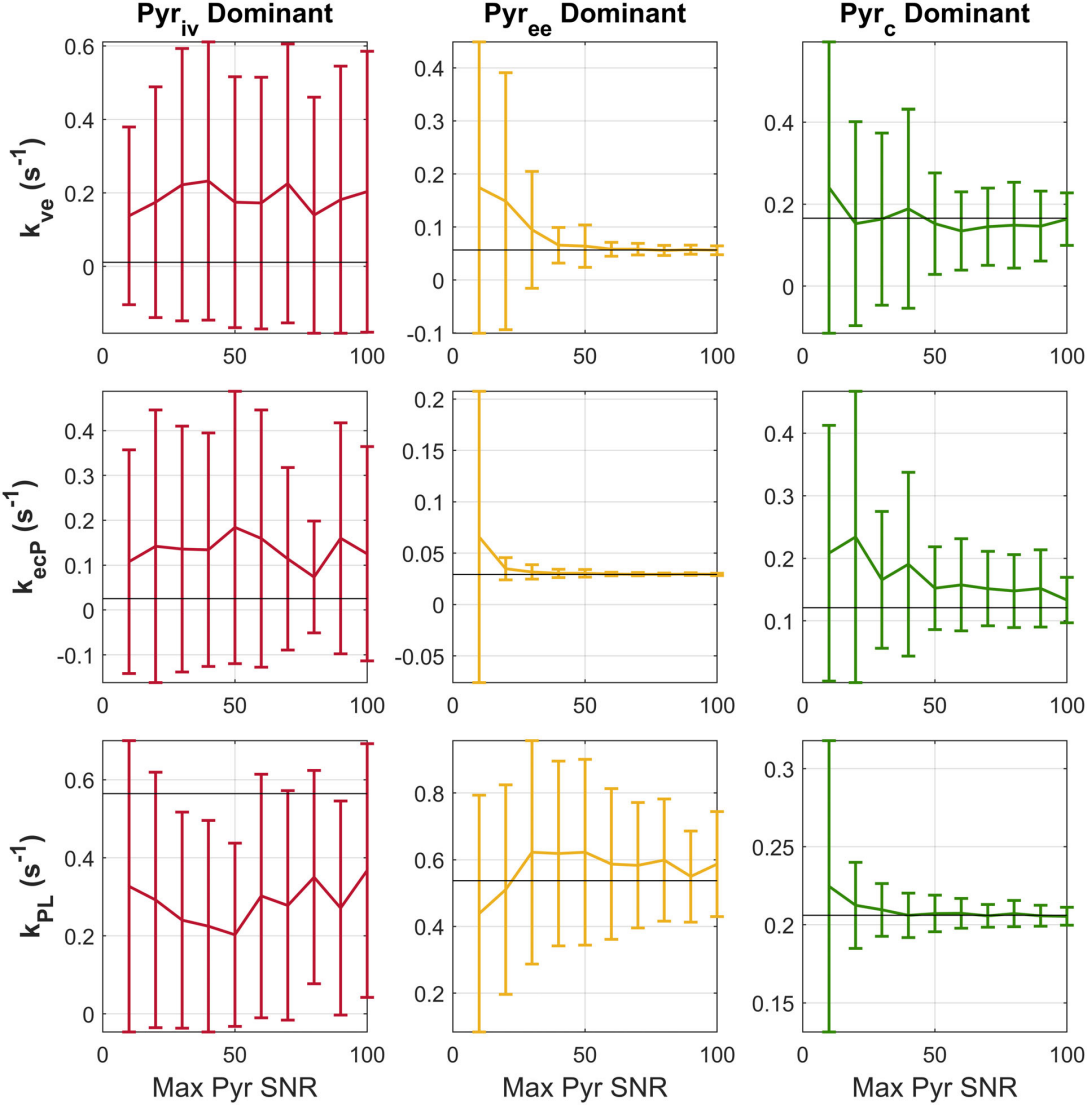
The accuracy and reproducibility of fitted PK parameters to noisy data is sensitive to the compartment which dominates the pyruvate signal. Synthetic data were generated with the 3PCs model for three sets of PK parameters (Table 1B). Reference parameter values are shown in horizontal black lines. When intravascular spins dominate pyruvate signal (red), standard deviation in all fitted parameters is high. When extravascular/extracellular spins dominate (yellow), $k_{\mathrm{ve}}$ and $k_{\mathrm{ecP}}$ estimates are reasonable, but fitted $k_{\mathrm{PL}}$ variance is still high. Only when intracellular spins dominate (green) is a reproducible estimate of $k_{\mathrm{PL}}$ possible under modest SNR conditions.

The accuracy of fitted kinetic rate constants was related to the physical compartment that contributed the most to the overall pyruvate signal. When pyruvate signal predominantly arose from intravascular spins, the uncertainty in all fitted kinetic rate constants was high. When extravascular/extracellular pyruvate spins instead dominated, it was possible to estimate pyruvate extravasation ($k_{\mathrm{ve}}$) and uptake ($k_{\mathrm{ecP}}$). Under this regime, it was still difficult to estimate intracellular metabolism, as the uncertainty on fitted $k_{\mathrm{PL}}$ was high. Accurate and reproducible measurements of intracellular metabolism were only achieved when intracellular pyruvate dominated the pyruvate signal (Figure 4). Otherwise, other processes were rate‐limiting and obscured the measurement of $k_{\mathrm{PL}}$. For an SNR of 20, the coefficient of variation of fitted $k_{\mathrm{PL}}$ was 1.26 for intravascular pyruvate dominance, 0.62 for extravascular/extracellular dominance, and 0.11 for intracellular dominance. This further supports the notion that underlying data (and the AUC values that may summarize them) does not provide direct insight about intracellular metabolism when extravasation or transport represents the predominant rate‐limiting step.

### 4.4 3PCs Model Framework Can Correct Calculated AUC Ratio for Different Choice of Echo Time Between Acquisitions

**FIGURE 5:**
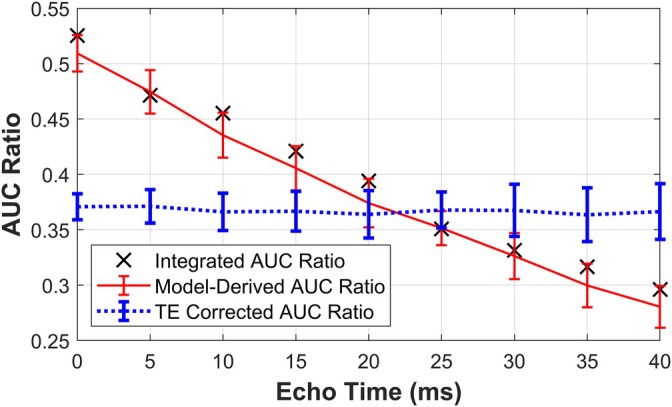
The AUC ratio is sensitive to changes in echo time (TE) but can be recalculated to only reflect changes in physiology. 50 sets of noisy, synthetic time curves were generated with the 3PCs model for a range of echo times. The integrated AUC ratio was calculated as the sum under the signal time curves. The model‐derived AUC ratio was calculated from fitted model parameters to recapitulate the integrated AUC ratio. Finally, the model‐derived AUC ratio was corrected for TE effects by recalculating with TE = 20 ms.

Most analyses assume that *T*_2_* and TE have negligible effects on metrics of metabolism, but changes in the acquisition parameters can affect relative signal levels and subsequent quantification [21]. In this work, the AUC ratio calculated using the traditional integration method decreased with increasing TE (Figure 5) since the *T*_2_* for lactate was assumed to be shorter than the *T*_2_* for pyruvate [20]. Through inclusion of $\mathrm{TE}/T_{2}^{*}$ effects in 3PCs, the AUC ratio can be recalculated using Equation (15), allowing adjustment of the measured AUC ratio to what would have been measured using a different set of acquisition parameters (Figure 5). The error in the model‐derived and corrected AUC ratios at an SNR of 20 was much lower than the error for individual fitted parameters (Figure 4). This style of correction is in principle also possible for other choices of acquisition parameters such as TR or flip angles.

### 4.5 The 3PCs Model Permits Identification of the Dominant Pyruvate Pool With Good Sensitivity and High Specificity

**FIGURE 6:**
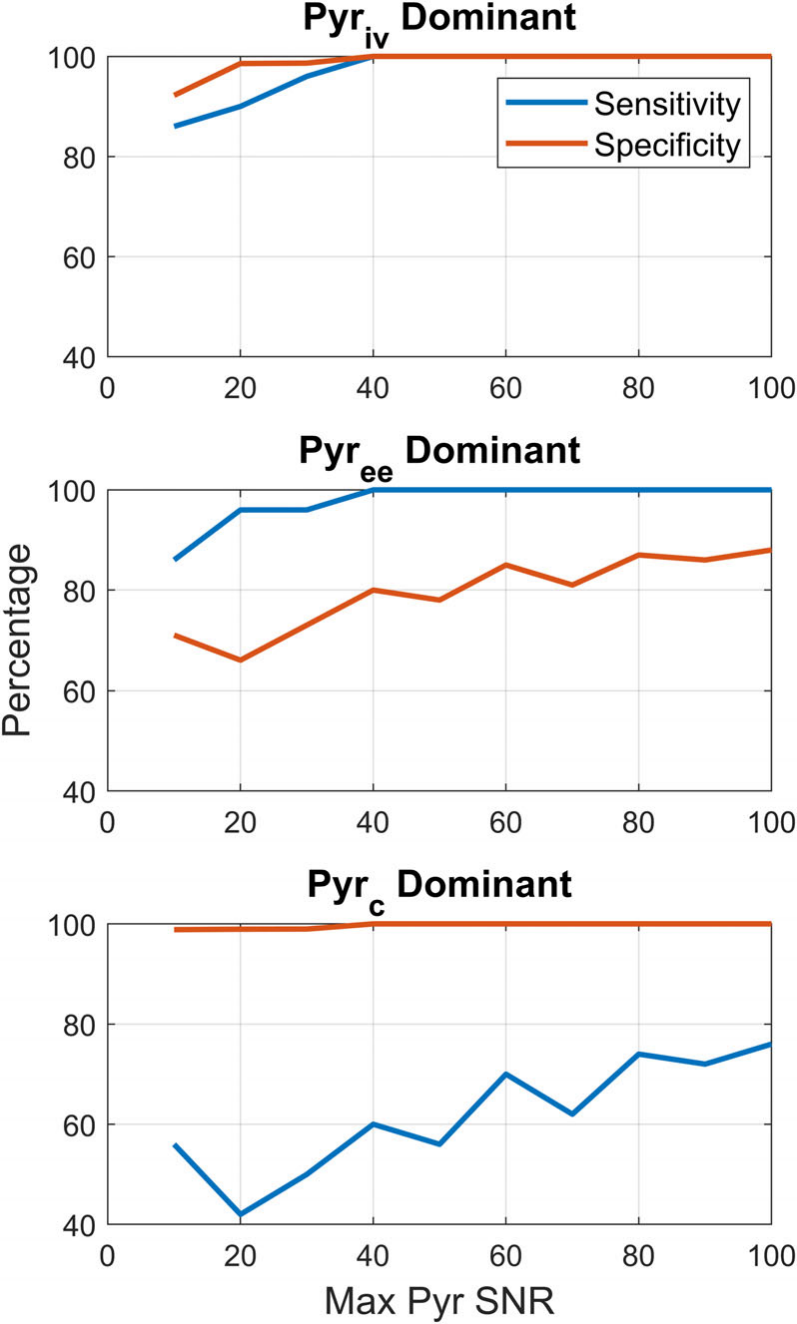
Sensitivity and specificity analysis of classification of the dominant pyruvate compartment. The dominant compartment was defined as the compartment that contributes most to the pyruvate signal. When intravascular pyruvate represented the dominant source (61.0%; see Table 1) of pyruvate signal, terms in the denominator of Equation (15) could identify intravascular pyruvate as the dominant source with at least 86% sensitivity and 92% specificity, even when the maximum pyruvate SNR was as low as 10. Dominant extravascular/extracellular pyruvate (65.5%) was identified with at least 86% sensitivity and 66% specificity, and dominant intracellular pyruvate (67.7%) was identified with at least 42% sensitivity and 98% specificity.

The specificity of the model to the dominant pyruvate compartment was near 100% for both the intravascular and intracellular cases even at a peak pyruvate SNR as low as 10 (Figure 6). Likewise, the sensitivity was greater than 85% for both the intravascular and extravascular/extracellular cases at low SNR. When the model incorrectly predicted the greatest source of the pyruvate, it was most likely to misattribute intracellular spins to the extravascular/extracellular compartment, resulting in a sensitivity as low as 40% for classifying intracellular pyruvate under low SNR conditions.

### 4.6 In Vivo Data Shows HP Pyruvate Mostly Arises From the Extravascular/Extracellular Compartment in Patients With Untreated Squamous Cell Carcinoma

**FIGURE 7:**
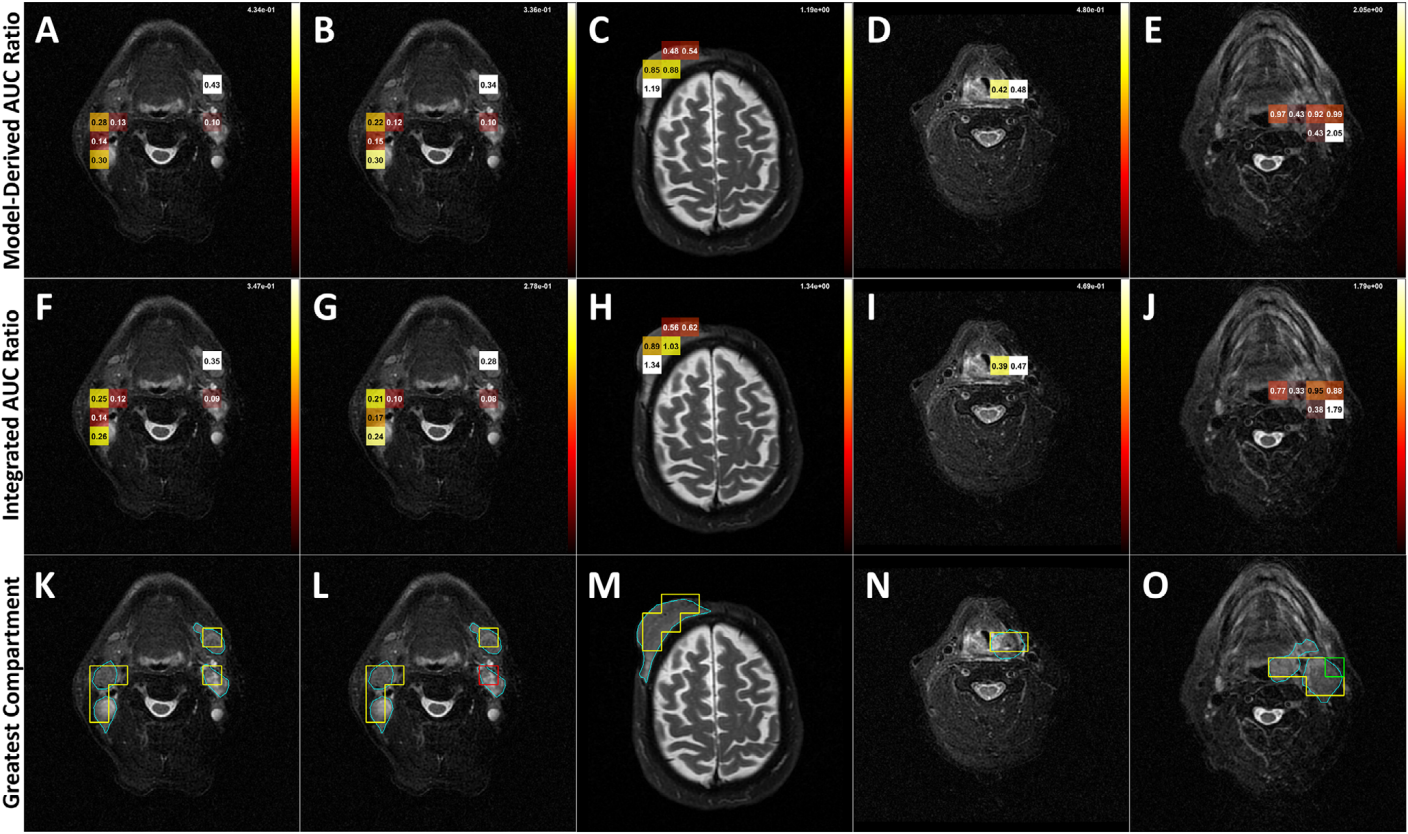
Overlay of the AUC ratio and relative pool size categorization on T2w reference images. Panels C, H and, M show data from a patient with cutaneous squamous cell carcinoma (SCC). All other patients had HPV‐associated oropharyngeal SCC. Panels A and B are two datasets from the same patient during a single imaging session. The relative size of terms in the denominator of Equation (15) can facilitate more specific interpretation of the AUC ratio according to Equations ((16), (17), (18)). Parametric analysis of the patient data reveals that in different voxels, the majority of signal from HP pyruvate may arise from intravascular (red), extravascular/extracellular space (yellow), and/or intracellular space (green). Tumor ROI were outlined in blue. For the 25 voxels analyzed, 23 were identified as extravascular/extracellular dominant.

The analytical expression of the AUC ratio was successfully applied in the analysis of five datasets (Figure 7), including one test–retest dataset (Figure 7A,B,F,G). Of the 74 voxels with high enough pyruvate SNR to be included in the analysis, intravascular pyruvate was the dominant compartment in three voxels, extravascular/extracellular pyruvate was dominant in 69 voxels, and intracellular pyruvate was dominant in two voxels.

A single patient was scanned twice during the same imaging session (Figure 7A,B). In the first scan, all voxels in the tumor ROI were identified as extravascular/extracellular dominant, while in the second scan one voxel was identified as intravascular dominant. This change could reflect slight physiological changes between scans, a slight change in patient position, the imperfect sensitivity and specificity of the method, or a combination of these factors.

### 4.7 Model‐Derived AUC Ratio Is Highly Correlated With the Integrated AUC Ratio

**FIGURE 8:**
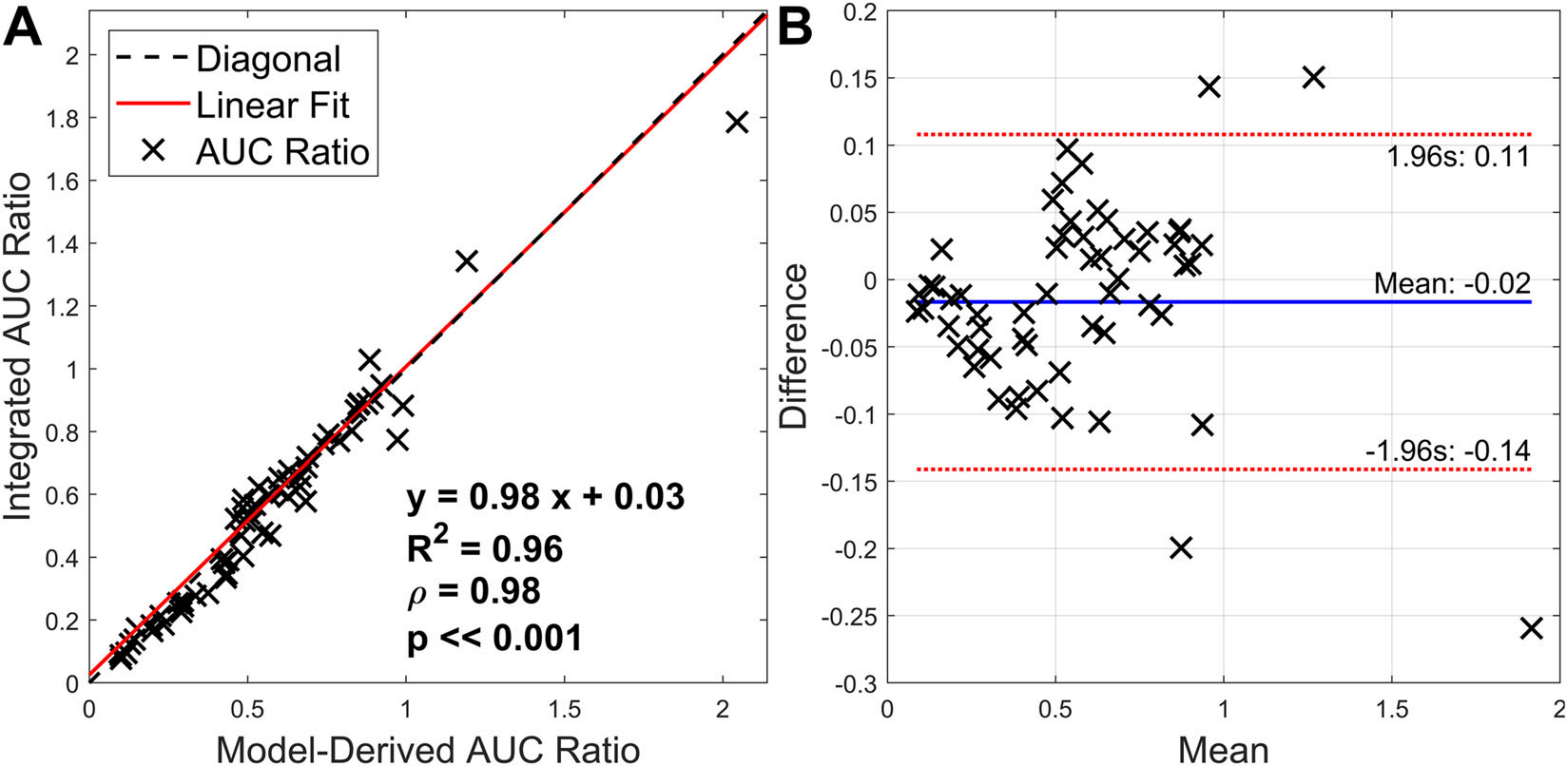
The correlation between integrated AUC ratio and the AUC ratio calculated from fitted PK parameters. (A) The AUC ratio calculated from fitted 3PCs parameters and Equation (15) shows high correlation (*ρ* = 0.98, *p* ≪ 0.001) with the AUC ratio calculated from the observed data by integrating the area under the pyruvate and lactate curves over time. (B) Bland Altman analysis of the difference between the integrated and model‐derived AUC ratios and their mean. The mean difference between the two metrics and the lines of 1.96 standard deviations of the differences are marked in blue and red respectively.

The AUC ratio calculated by integrating the observed signal time curves (integrated AUC ratio) exhibited strong correlation to the AUC ratio calculated from fitted PK parameters (Figure 8A) with a Pearson correlation coefficient of 0.98. The slope for the linear fit relationship was near diagonal with an *R* ^2^ value of 0.96. Bland–Altman analysis (Figure 8B) demonstrated that the model‐derived AUC ratio only slightly underestimates the integrated AUC ratio as evidenced by the negative mean value. The standard deviation of the difference was 0.12 over a range of mean values up to ∼1.9. The near equivalence of the integrated and model‐derived AUC ratios suggests that the 3PCs model adequately fits the data and that Equation (15) accurately represents the AUC ratio.

## 5 Discussion and Conclusions

Analysis of HP pyruvate MRI is currently limited by the lack of a standardized metric for the rate of aerobic glycolysis, which obscures the interpretation acquired data. In this work, we have critically examined the lactate AUC to pyruvate AUC ratio as a metric of tumor metabolism and developed a novel approach to aid in its interpretation. The AUC ratio has been described analytically in terms of acquisition parameters ($\theta_{\mathrm{Pyr}},\theta_{\mathrm{Lac}}$, *TR*, *TE*) and PK parameters for a specialized model (3PCs) with three physical compartments and two chemical pools. The expression for the AUC ratio is independent of the VIF, provided the bolus arrives after the start of data acquisition to ensure that no signal is present at t=0 [15]. In principle, this analysis is possible with a wide range of compartmental models.

Simplified expressions for the AUC ratio identify its dependence on different model parameters when the HP pyruvate signal is dominated by intravascular, extracellular/extravascular, or intracellular pyruvate pools. In a noiseless environment, the fitted parameters from the simplified model were reasonably well associated with the relevant full 3PC reference model parameters for the three parameter sets examined (Table 1). In the presence of noise, however, the precision of fitted parameters varies and is dependent on the underlying physiology (Figure 4). These results show that it is difficult to estimate specifically intracellular metabolism (*k*_*PL*_) except when intracellular pyruvate is the dominant pool, even under highly favorable conditions with few unknown parameters. This further suggests that when intracellular pyruvate is not the dominant pool of HP pyruvate, the underlying data, and hence the AUC ratio that summarizes the data, would not directly represent intracellular metabolism.

Taken together with Equations ((16), (17), (18)), Figure 3 suggests a connection between the rate‐limiting step in lactate production and the physical compartment with the most HP pyruvate spins. Here again, the AUC ratio gives the most direct insight into intracellular metabolism (*k*_*PL*_) when intracellular pyruvate is the dominant pool. Relative pool sizes, and thus the relative importance of different PK parameters to the AUC ratio, can be calculated from the denominator of Equation (15) using parameter values derived by fitting the 3PCs model to observed data. Pool sizes that are estimated this way may be more reproducible than individual model parameters. Using the 3PCs model and parameter‐minimized least squares fitting, we observe greater than 98% specificity for determining when intracellular metabolism is the rate‐limiting step in lactate production (Figure 6). While the sensitivity of the method to identifying dominance of the intracellular pyruvate pool is modest, the specificity is high and the sensitivity and specificity of the method to identifying dominance of other compartments is good even at relatively low SNR (Figure 6). Approaches from past studies to optimize imaging strategies for robustness of fitted PK parameters [21, 25, 26] may be combined in future work with the mathematical framework here developed to increase the sensitivity of AUC ratio to intracellular metabolism.

Here we demonstrated the feasibility of kinetic modeling to provide additional mechanistic insight about the AUC ratio without direct comparison of individual model parameters. In the patient datasets analyzed, the dominant source of pyruvate was primarily the extravascular/extracellular compartment (Figure 7), indicating that pyruvate transport (*k*_*ecP*_) was a significant rate‐limiting step for lactate production in most voxels. This framework could be used to facilitate grouping of voxels with similar physiological status for subsequent analysis, or to identify the appropriate level of model complexity if further PK analysis is pursued. For example, a full 3PC model would not be appropriate when transport is known to be the primary rate‐limiting step of HP lactate production. Further study and much more HP MRI data, including correlative pathology, is needed to fully establish the potential role for this framework in aiding the interpretation of the AUC ratio and for enhancing analysis of HP MRI data. Although some studies report strong correlation between the AUC ratio and $k_{\mathrm{PL}}$, others have found unfavorable variability [16, 27]. Even while changes in the AUC ratio may not always be strictly attributable to changes in intracellular metabolism, it remains a useful surrogate for characterization of HP MRI signal evolution and quantifying tumor aggression. Several studies have identified cancers in which lactate production is critically rate limited by pyruvate transport [28, 29]. In these cases, overall lactate production may still be a useful indicator of tumor aggression when MCTs are upregulated by cancer cells [30]. The AUC ratio may be influenced by several potentially rate‐limiting steps, all of which may be affected by disease and treatment: extravasation of pyruvate from the vasculature [12, 31], transport of pyruvate across the cell membrane [10, 30], or the intracellular chemical conversion of pyruvate to lactate [32]. Identification of the dominant source of HP pyruvate signal using this approach can provide additional mechanistic insight into the dominant factors that influence lactate production.

This framework also facilitates direct comparisons of AUC ratios between sites using different acquisition parameters. We demonstrated that TE effects can be corrected (Figure 5) by recalculating the AUC ratio via Equation (15) using fitted PK model parameters and the desired value for TE. In principle, the same methodology could also allow correction for inconsistent TR, flip angle schemes, and slice profiles, but further research is necessary to ensure that propagation of error from incompletely known parameters remains lower than differences induced by acquisition parameters. While the precision of individual fitted parameters depends on physiology and on the SNR of data, the calculated AUC ratio from fitted parameters from several in vivo studies was robust (Figure 8).

In conclusion, the ratio of the lactate AUC to the pyruvate AUC may reflect intracellular metabolism, pyruvate uptake across the cell membrane, or pyruvate extravasation, depending on whether the dominant source of HP pyruvate signal arises from intracellular, extracellular/extravascular, or intravascular pools of HP pyruvate, respectively. This framework enables determination of the dominant source of HP pyruvate signal for a given voxel and thus improves our ability to interpret the AUC ratio and changes that may be induced by disease progression or response to treatment.

## Funding

This research was supported in part by funding from the National Cancer Institute (R01CA211150, R01CA280980, and P30CA016672) and the National Institute of Biomedical Imaging and Bioengineering (R01EB032376) of the National Institutes of Health. The content is solely the responsibility of the authors and does not necessarily represent the official views of the sponsors.

## Conflicts of Interest

Stephen Y. Lai receives research funding from the National Institutes of Health and serves as a medical affairs consultant for Cardinal Health. James A. Bankson receives research funding from the National Institutes of Health and from Siemens Medical Solutions USA Inc. He serves as a member of the Scientific Advisory Board for NVision Imaging Technologies GmbH, and he owns shares of NVision. Matthew E. Merritt receives research funding from the National Institutes of Health.

## Supporting information


**Data S1:** Derivation of the parameterized AUC ratio from the full three‐compartment (3PC) model. The AUC ratio can be derived by application of the Laplace transform to the full system of coupled differential equations that describe 3PC. The resulting equation is significantly more complex than the parameterized AUC ratio derived from the 3PCs model.

## Data Availability

Data and scripts used for simulations and analysis are available from the following link: https://github.com/mda‐mrsl/c13‐pyr‐auc‐phys‐interp/tree/61f686c. Additional data are available from the corresponding author upon reasonable request.
