# Supplementary material for: Physiological Interpretation of the Lactate to Pyruvate AUC Ratio for Hyperpolarized [1‐13C]‐Pyruvate Studies
Source: Magn Reson Med. 2026 Feb 27;96(1):247–58. doi: 10.1002/mrm.70309 (PMC13156448; doi:10.1002/mrm.70309)
Supplement: Supplementary file 1 — Data S1: Derivation of the parameterized AUC ratio from the full three‐compartment (3PC) model. The AUC ratio can be derived by application of the Laplace transform to the full system of coupled differential equations that describe 3PC. The resulting equation is significantly more complex than the parameterized AUC ratio derived from the 3PCs model. [file MRM-96-247-s001.docx]

Calculating the AUC Ratio from the Full Three-Compartment Model:

In addition to perfusion and metabolism, lactate production may also be influenced by the rate of pyruvate uptake across the cell membrane. The 2PC model cannot distinguish between the effects of uptake and metabolism because it does not consider the space between cells in which the enzymes responsible for catalyzing the reaction between pyruvate and lactate are not present. This motivates the inclusion of two extravascular compartments: the extravascular/extracellular (*ee)* compartment and the intracellular (*c*) compartment. Chemical conversion only takes place in the intracellular compartment.

The three-physical-compartment (3PC) model consists of the intravascular, extravascular/extracellular, and intracellular compartments. It has the advantage of being able to account for the rate of uptake of pyruvate into the cell, $k_{ecp},$ and the rate of lactate export out of the cell, $k_{ecl}$. The differential equations for the 3PC model are as follows:

$$\frac{\partial}{\partial t}\left[ Pyr_{ee}\left( t \right) \right]=-\left( \frac{k_{ve}}{v_{ee}}+\frac{k_{ecp}}{v_{ee}}+R_{1,Pyr}+\frac{1-cos\theta_{Pyr}}{TR} \right)\left[ Pyr_{ee}\left( t \right) \right]+\frac{k_{ecp}}{v_{ee}}\left[ Pyr_{c}\left( t \right) \right]+\frac{k_{ve}}{v_{ee}}\left[ Pyr_{iv}\left( t \right) \right]$$

$$\begin{aligned} =\alpha_{Pee}\left[ Pyr_{ee}\left( t \right) \right]+\frac{k_{ecp}}{v_{ee}}\left[ Pyr_{c}\left( t \right) \right]+\frac{k_{ve}}{v_{ee}}\left[ Pyr_{iv}\left( t \right) \right]\#\left[ S1 \right] \end{aligned}$$

$$\frac{\partial}{\partial t}\left[ Lac_{ee}\left( t \right) \right]=-\left( \frac{k_{ve}}{v_{ee}}+\frac{k_{ecl}}{v_{ee}}+R_{1,Lac}+\frac{1-cos\theta_{Lac}}{TR} \right)\left[ Lac_{ee}\left( t \right) \right]+\frac{k_{ecl}}{v_{ee}}\left[ Lac_{c}\left( t \right) \right]$$

$$\begin{aligned} =\alpha_{Lee}\left[ Lac_{ee}\left( t \right) \right]+\frac{k_{ecl}}{v_{ee}}\left[ Lac_{c}\left( t \right) \right]\#\left[ S2 \right] \end{aligned}$$

$$\frac{\partial}{\partial t}\left[ Pyr_{c}\left( t \right) \right]=\frac{k_{ecp}}{v_{c}}\left[ Pyr_{ee}\left( t \right) \right]-\left( \frac{k_{ecp}}{v_{c}}+k_{pl}+R_{1,Pyr}+\frac{1-cos\theta_{Pyr}}{TR} \right)\left[ Pyr_{c}\left( t \right) \right]+k_{lp}\left[ Lac_{c}\left( t \right) \right]$$

$$\begin{aligned} =\frac{k_{ecp}}{v_{c}}\left[ Pyr_{ee}\left( t \right) \right]+\alpha_{Pc}\left[ Pyr_{c}\left( t \right) \right]+k_{lp}\left[ Lac_{c}\left( t \right) \right]\#\left[ S3 \right] \end{aligned}$$

$$\frac{\partial}{\partial t}\left[ Lac_{c}\left( t \right) \right]=\frac{k_{ecl}}{v_{c}}\left[ Lac_{ee}\left( t \right) \right]-\left( \frac{k_{ecl}}{v_{c}}+k_{lp}+R_{1,Lac}+\frac{1-cos\theta_{Lac}}{TR} \right)\left[ Lac_{c}\left( t \right) \right]+k_{pl}\left[ Pyr_{c}\left( t \right) \right]$$

$$\begin{aligned} =\frac{k_{ecl}}{v_{c}}\left[ Lac_{ee}\left( t \right) \right]+\alpha_{Lc}\left[ Lac_{c}\left( t \right) \right]+k_{pl}\left[ Pyr_{c}\left( t \right) \right]\#\left[ S4 \right] \end{aligned}$$

Here $\alpha_{Pee}$, $\alpha_{Lee}$, $\alpha_{Pc}$, and $\alpha_{Lc}$ are the compartmental signal loss terms for extravascular/extracellular pyruvate, extravascular/extracellular lactate, intracellular pyruvate, and intracellular lactate.

Eqs. [1]-[4] are a system of linear, coupled, first order differential equations with five degrees of freedom and four constraints. The AUC ratio of the 3PC model is given by

$$\begin{aligned} \frac{AUC_{Lac}}{AUC_{Pyr}}=\frac{sin\theta_{Lac}}{sin\theta_{Pyr}}\cdot\frac{v_{c}\bar{Lac_{c}}\left( s=0 \right)+v_{ee}\bar{Lac_{ee}}\left( s=0 \right)}{v_{b}\bar{Pyr_{iv}}\left( s=0 \right)+v_{ee}\bar{Pyr_{ee}}\left( s=0 \right)+v_{c}\bar{Pyr_{c}}\left( s=0 \right)}\#\left[ S5 \right] \end{aligned}$$

Following the same procedure used to solve the 2PC model, the AUC of each metabolite in each compartment can be found in terms of the VIF by rearranging the Laplace transform of the system.

$$\frac{AUC_{Lac}}{AUC_{Pyr}}=\frac{sin\theta_{Lac}}{sin\theta_{Pyr}}\cdot\frac{k_{ecp}}{v_{c}}\frac{k_{ve}}{v_{ee}}\left( k_{ecl}-\alpha_{Lee}v_{c} \right)$$

$$\div\left( \alpha_{Pee}\alpha_{Pc}\alpha_{Lee}\alpha_{Lc}-\alpha_{Pc}\alpha_{Lee}\alpha_{Lc}k_{ve}-\alpha_{Lee}\alpha_{Lc}\frac{k_{ecp}}{v_{ee}}\frac{k_{ecp}}{v_{c}}v_{b}+\alpha_{Lee}\alpha_{Lc}\frac{k_{ve}}{v_{ee}}k_{ecp} \right.$$

$$-\alpha_{Pee}\alpha_{Lee}k_{pl}k_{lp}v_{b}+\alpha_{Lee}k_{pl}k_{lp}k_{ve}-\alpha_{Pee}\alpha_{Pc}\frac{k_{ecl}}{v_{ee}}\frac{k_{ecl}}{v_{c}}v_{b}-\alpha_{Pic}\frac{k_{ecl}}{v_{ee}}\frac{k_{ecl}}{v_{c}}k_{ve}$$

$$\begin{aligned} \left. +\frac{k_{ecl}}{v_{ee}}\frac{k_{ecl}}{v_{c}}\frac{k_{ecp}}{v_{ee}}\frac{k_{ecp}}{v_{c}}v_{b}-\frac{k_{ecl}}{v_{ee}}\frac{k_{ecl}}{v_{c}}\frac{k_{ve}}{v_{ee}}k_{ecp} \right)\#\left[ S6 \right] \end{aligned}$$
